# Pleural Effusions Requiring Thoracocentesis Are Associated With Baseline Lung Allograft Dysfunction and Mortality in Lung Transplant Recipients

**DOI:** 10.1111/ctr.70234

**Published:** 2025-08-22

**Authors:** Michael Gerckens, Nicole Weiss, Daria Khmelovska, Alexander Richard, Mathias Klemm, Philipp Plohmann, Paola Arnold, Tobias Veit, Jürgen Barton, Teresa Kauke, Christian Schneider, Sebastian Michel, Michael Irlbeck, Ali Önder Yildirim, Jürgen Behr, Nikolaus Kneidinger, Carlo Mümmler

**Affiliations:** ^1^ Department of Medicine V LMU University Hospital, LMU Munich, Comprehensive Pneumology Center (CPC), Member of the German Center of Lung Research (DZL) Munich Germany; ^2^ Institute of Lung Health and Immunity (LHI), Comprehensive Pneumology Center (CPC) Helmholtz Munich, Member of the German Center of Lung Research (DZL) Munich Germany; ^3^ Department of Medicine I LMU University Hospital LMU Munich Munich Germany; ^4^ Department of Medicine III LMU University Hospital LMU Munich Munich Germany; ^5^ Division of Thoracic Surgery LMU University Hospital LMU Munich Munich Germany; ^6^ Department of Cardiac Surgery LMU University Hospital LMU Munich Munich Germany; ^7^ Department of Anaesthesiology LMU University Hospital LMU Munich Munich Germany; ^8^ Institute of Experimental Pneumology, LMU University Hospital Ludwig Maximilians University of Munich Munich Germany; ^9^ Department of Internal Medicine, Division of Respiratory Medicine, Lung Research Cluster Medical University of Graz Graz Austria

**Keywords:** baseline lung allograft dysfunction, BLAD, lung transplantation, mortality, pleural effusions, pleural, survival

## Abstract

**Background:**

Pleural effusions of unknown etiology have been demonstrated to be associated with poor prognosis in lung allograft recipients. We aimed to identify novel risk factors for pleural effusions after lung transplantation (LTX) and to shed light on their association with allograft function and survival, differentiating early and late pleural effusions after LTX.

**Methods:**

We performed a retrospective study of all LTX recipients transplanted at the LMU Klinikum Munich from 2013 to 2018. We recorded all pleural effusions requiring thoracocentesis and analyzed the corresponding clinical data. A total of 426 pleural effusions in 369 lung allograft recipients with a median follow‐up time of 6.9 years were analyzed.

**Results:**

Both early (<90 days after LTX) and late pleural effusions (>90 days after LTX) were associated with increased mortality, with a strong mortality risk for late pleural effusions (HR 4.0). Increased mortality in patients with early pleural effusions might be mediated by a higher risk for baseline lung allograft dysfunction (BLAD). Early pleural effusions were associated with underlying obstructive disease, relative donor organ undersizing and clamshell thoracotomy. Notably, (partial) resection of the parietal pleura during LTX was not associated with pleural effusions.

**Conclusions:**

This study underlines the importance of pleural effusions after LTX. All pleural effusions were associated with increased mortality, while only early pleural effusions were associated with BLAD. The mechanisms linking pleural effusions to BLAD and to higher mortality remain unknown and will be investigated in future, prospective cohorts.

**Abbreviations**
95% CI: 95% confidence interval
BLAD: baseline lung allograft dysfunction
BMI: body mass index
CLAD: chronic lung allograft dysfunction
DLTX: double lung transplantation
FEV_1_
: forced expiratory volume in 1 s
FVC: forced vital capacity
HR: hazard ratio
IQR: interquartile range
LTX: lung transplantation
PFT: pulmonary function testing
RV: residual volume
SLTX: single lung transplantation

## 1 Introduction

Pleural abnormalities and pleural effusions occur frequently in lung transplant (LTX) recipients and have been associated with poor patient outcomes after LTX [1, 2]. Recent analysis demonstrated that the majority of pleural effusions after LTX are of unclear origin [1] and that the high mortality of LTX pleural effusions is not due to a subsequent development of chronic lung allograft dysfunction (CLAD) [1].

Previous studies have indicated that early and late pleural effusions might have different characteristics [1]. Usually, early occurring pleural effusions are regarded as benign, with initially highly neutrophilic exudates, that become more lymphocytic over time [3]. However, the influence of transplant surgery on the incidence of early pleural effusions as well as their impact on subsequent lung function development remains unknown. Besides CLAD, another emerging concept that predicts survival after LTX is baseline lung allograft dysfunction (BLAD) [4]. BLAD is defined as the failure to achieve a certain lung function threshold after transplantation [4, 5]. Among others, BLAD has been associated with donor‐recipient size mismatch [5, 6]. Unclear pleural effusions after LTX have also been shown to be associated with donor‐recipient size mismatch [1, 2]. Thus, it is possible that the common denominator by which size mismatching impacts lung function is an underlying process leading to the subsequent formation of pleural effusions.

Here, we conducted a single‐center, retrospective study of 369 patients to analyze associations of pleural effusions with BLAD and to identify novel risk factors specific for early and late pleural effusions after LTX. We set a specific focus on lung function trajectories and BLAD while differentiating early and late pleural effusions after LTX.

## 2 Methods

### 2.1 Ethics

This study was conducted in accordance with the Declaration of Helsinki and approved by the Ethics Committee of the Ludwig‐Maximilians‐University of Munich (#21‐0020). Broad informed consent was obtained from all participants at the time of transplant evaluation.

### 2.2 Patient Cohort

We retrospectively reviewed all adult single and double lung allograft recipients (*n* = 369) transplanted at our center between 2013 and 2018. Patients were followed up until death, retransplantation, or until May 1^st^, 2024. As the definitions of BLAD and CLAD require at least two lung function tests more than 21 days apart, patients who did not meet this criterion were excluded from the analysis of lung function trajectories (*n* = 45), leaving a cohort of 324 patients for the analysis of BLAD and CLAD endpoints.

### 2.3 Retrospective Analysis of Pleural Effusion Thoracocentesis

All patient documentation was reviewed for LTX recipients with clinically relevant pleural effusions that underwent thoracocentesis. Indication for pleural effusion thoracocentesis was at the discretion of the treating physician. Reasons for pleural effusion thoracocentesis could include pleural effusion associated dyspnea, the diagnostic work‐up of an unclear pleural effusion, the relief of a pleural effusion in the case of loss of lung function, a large‐sized pleural effusion, and/or atelectasis associated with pleural effusions. To ensure a valid, well‐defined endpoint, only pleural effusions that were therapeutically drained were included, as we considered this to be less biased compared to analyzing pleural effusion occurrence or disappearance on imaging. Pleural effusions that were drained up to 90 days after transplantation were classified as “early,” while pleural effusions that were drained after 90 days were classified as “late.” Patients who underwent both early and late pleural effusion thoracentesis were included in both groups for respective analyses. Relevant clinical parameters were collected per pleural effusion.

### 2.4 Standard LTX Management

Treatment regimens and standardized follow‐up of LTX recipients at LMU Munich were performed as previously described [7].

### 2.5 Lung Function Testing

Lung function testing was performed by spirometry and body plethysmography (Jäger, Würzburg, Germany) according to current ERS/ATS guidelines [8]. Routine pulmonary function tests at our center were performed quarterly and when clinically indicated. GLI reference equations were used according to Stanojevic et al. [9].

### 2.6 Baseline Allograft Dysfunction and CLAD

Baseline lung function was calculated with percentage predicted of FEV1 (forced expiratory volume in 1 s) and FVC (forced vital capacity) using the Global Lung Initiative (GLI) reference equations based on the allograft recipient characteristics. For DLTX (double lung transplantation) recipients, BLAD was defined as failure to reach both FEV1% and FVC% >80% predicted on at least two consecutive tests more than 21 days apart [4]. For SLTX recipients, we defined baseline allograft dysfunction (BLAD) as failure to reach both FEV1% and FVC% >60% predicted on at least two consecutive tests more than 21 days apart [5]. CLAD was diagnosed as a persistent FEV1 decline to less than 80% of the baseline FEV1 that persisted for more than 3 months and was not attributable to alternative causes, according to current ISHLT guidelines [10].

### 2.7 Interpolation of Lung Function Trajectories

For time course plotting, pulmonary function testing (PFT) data were imputed by linear interpolation to achieve daily estimations of average lung function of the study population and respective subgroups [11]. Imputed PFT data were left‐ and right‐censored from the first PFT measurement post‐transplantation to the last recorded PFT measurement.

### 2.8 Outcomes

The primary endpoint, “allograft survival,” was defined as time to death or retransplantation.

### 2.9 Statistical Analysis

For descriptive statistics, data are presented as median and range. Interferential statistics were performed using Mann‐Whitney‐U for continuous variables, not assuming normal distribution. For contingency table analysis, the Chi‐squared test was used. Right‐censored time‐to‐event analysis was analyzed using the Cox regression model. Associations between status post pleural effusion thoracocentesis and survival were assessed using time‐varying Cox regression analysis. Data analysis, including Cox regressions and Kaplan‐Meier estimators, was performed with Python 3.10 and the packages numpy (v1.22.3), pandas (v1.4.2), matplotlib (v3.5.2), seaborn (v0.11.2), lifelines (v0.27.0), and scipy (v1.8.0). Additional statistical analysis was performed with GraphPad Prism 8 (GraphPad Software, San Diego, CA). Used statistical tests and models are indicated in the respective tables and figures.

## 3 Results

### 3.1 Study Cohort

We analyzed the follow‐up of all 369 lung allograft recipients transplanted at our center at LMU Munich between 2013 and 2018. In this cohort, altogether 426 pleural effusions requiring thoracocentesis were recorded in 156 lung allograft recipients, while 213 lung allograft recipients did not need pleural effusion thoracocentesis (Figure S1). Sixty‐four patients had bilateral pleural effusion thoracocentesis, 45 patients only had left‐sided pleural effusion thoracocentesis, and 47 patients only had right‐sided pleural effusion thoracocentesis. In 57 patients, only one pleural effusion thoracocentesis was performed, in 99 patients multiple pleural effusion thoracocenteses were performed. Only for the analysis of lung function‐related endpoints like CLAD and BLAD, patients with less than two lung function measurements at least 21 days apart were excluded (*n* = 45), leaving a cohort of 324 lung allograft recipients. Median follow‐up was 6.9 years.

### 3.2 Baseline Characteristics of Patients Requiring Early and Late Pleural Effusion Thoracocentesis

Baseline characteristics differed between patients with and without early pleural effusion thoracocentesis (up to 90 days after LTX) as LTX recipients with early pleural effusions thoracocentesis were more frequently operated by clamshell procedure (*p* = 0.004), had lower BMI (body mass index) at transplantation (*p* = 0.006) and were more frequently transplanted directly from an ICU stay (*p* = 0.04) (Table 1). Baseline characteristics did not differ between patients with and without late pleural effusion thoracocentesis (more than 90 days after LTX) (Table 2).

**TABLE 1:** Comparison of patients with early pleural effusion thoracocentesis compared to patients without early effusion thoracocentesis after lung transplantation.

Characteristics	Patients with early effusion thoracocentesis	Patients without early effusion thoracocentesis	*p*
Number of patients	118	251	
Time from transplantation to pleural effusion thoracocentesis, MEDIAN (IQR, days)	26 (19–38)	−	
Transplant type			
Single LTX, *N* (%)	36 (31%)	87 (35%)	0.48 ^a^
Double LTX, *N* (%)	82 (69%)	164 (65%)	0.48 ^a^
Operating procedure			
Clamshell, *N* (%)	56 (47%)	72 (29%)	<0.001^a^
Anterior thoracotomy, *N* (%)	62 (53%)	175 (71%)	<0.001^a^
Partial pleural decortication, *N* (%)	43 (36%)	75 (30%)	0.23^a^
Underlying disease			
interstitial lung disease, *N* (%)	52 (44%)	137 (55%)	0.19^a^
cOPD, *N* (%)	39 (33%)	61 (24%)	
Bronchiectasis, *N* (%)	19 (16%)	29 (12%)	
PH, *N* (%)	3 (3%)	11 (4%)	
Other, *N* (%)	5 (4%)	13 (5%)	
Recipient characteristics			
Sex, *N* (%) female patients	65 (56%)	112 (45%)	0.07^a^
Age at LTX, median (IQR)	57 (42–62)	56 (48–62)	0.84^b^
BMI AT LTX, median (IQR)	22 (19–25)	23 (20–26)	0.006^b^
ICU stay at time of transplantation, *N* (%)	18 (15%)	20 (8%)	0.04^a^
Donor characteristics			
Donor age (years), median (IQR)	54 (39–60)	48 (35–60)	0.09^b^
D/R sex mismatch	31 (26%)	49 (19%)	0.17^a^
Donor smoker	43 (36%)	114 (45%)	0.21^a^
Patient outcomes			
CLAD, *N* (%)	25 (21%)	62 (25%)	0.79^a^
BLAD, *N* (%)	55 (47%)	99 (39%)	0.05^a^
Deaths, *N* (%)	58 (49%)	107 (43%)	0.01^c^

*Note:* Comparison of patients with early pleural effusion thoracocentesis (*n* = 118) (up to 90 days) compared to patients without early effusion thoracocentesis (*n* = 251) after lung transplantation.

^a^ Chi‐square/Fisher exact test.

^b^ Unpaired Mann‐Whitney test for non‐parametric testing.

^c^ Log‐rank test.

**TABLE 2:** Comparison of patients with late pleural effusion thoracocentesis compared to patients without late effusion thoracocentesis after lung transplantation.

Characteristics	Patients with late effusion thoracocentesis	Patients without late effusion thoracocentesis	*p*
Number of patients	77	292	
Time from transplantation to pleural effusion thoracocentesis, MEDIAN (IQR, days)	225 (129–601)	−	
Transplant type			
Single LTX, *N* (%)	27 (35%)	96 (33%)	0.79^a^
Double LTX, *N* (%)	50 (65%)	196 (67%)	0.79^a^
Operating procedure			
Clamshell, *N* (%)	24 (31%)	104 (36%)	0.50^a^
Anterior thoracotomy, *N* (%)	53 (68%)	184 (62%)	0.53^a^
Partial pleural decortication, *N* (%)	23 (30%)	95 (33%)	0.68^a^
Underlying disease			
interstitial lung disease, *N* (%)	35 (45%)	154 (53%)	0.52^a^
cOPD, *N* (%)	27 (35%)	73 (25%)	
Bronchiectasis, *N* (%)	9 (12%)	39 (13%)	
PH, *N* (%)	3 (4%)	11 (4%)	
Other, *N* (%)	3 (4%)	15 (5%)	
Recipient characteristics			
Sex, *N* (%) female patients	34 (44%)	143 (49%)	0.52^a^
Age at LTX, median (IQR)	58 (49–63)	55 (47–62)	0.09^b^
BMI at LTX, median (IQR)	23 (20–26)	23 (20–26)	0.82^b^
ICU stay at time of transplantation, *N* (%)	5 (6%)	33 (11%)	0.29^a^
Donor characteristics			
donor age (years), median (IQR)	52 (40‐59)	49 (35‐60)	0.29^b^
D/R sex mismatch	21 (27%)	59 (20%)	0.21^a^
Donor smoker	32 (42%)	125 (43%)	0.53^a^
Patient outcomes			
CLAD, *N* (%)	19 (25%)	68 (24%)	0.76^a^
BLAD, *N* (%)	37 (56%)	117 (45%)	0.13^a^
Deaths, *N* (%)	48 (62%)	117 (40%)	**0.05** ^c^

*Note:* Comparison of patients with late pleural effusion thoracocentesis (*n* = 77) (after 90 days) compared to patients without late effusion thoracocentesis (*n* = 292) after lung transplantation.

^a^ Chi‐Square/Fisher exact test.

^b^ Unpaired Mann‐Whitney test for non‐parametric testing.

cLog‐rank test.

### 3.3 Late, but Not Early, Pleural Effusions After LTX Are Associated With Mortality

**FIGURE 1:**
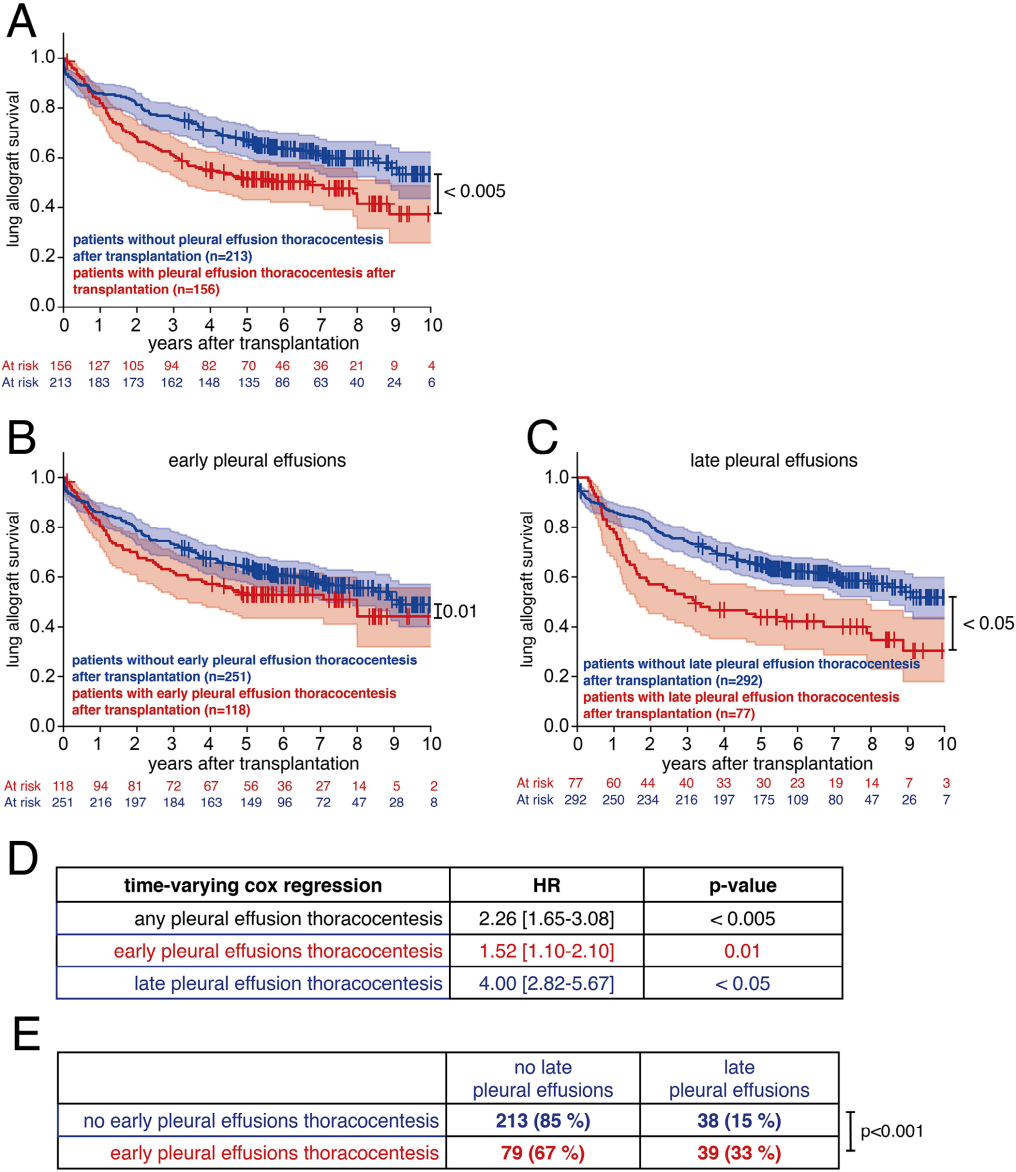
Late, but not early, pleural effusions requiring thoracocentesis after LTX are associated with mortality. (A) Kaplan Meier Plot illustrating allograft survival in patients with (red) and without (blue) pleural effusions that required thoracocentesis after lung transplantation. (B) Kaplan Meier Plot illustrating allograft survival in patients with early (red) and without early (blue) pleural effusions that required thoracocentesis after lung transplantation. (C) Kaplan Meier Plot illustrating survival in patients with late (red) and without late (blue) pleural effusions that required thoracocentesis after lung transplantation. (D) Cox regression analysis with status post thoracocentesis as a time‐varying risk factor. (E) Contingency table, displayed as a cross table, of patients with/without early and with/without late pleural effusions thoracocentesis. (D) Cox regression and (E) Fisher exact test.

LTX recipients who required pleural effusion thoracocentesis after transplantation displayed decreased lung allograft survival compared to LTX recipients without pleural effusions (*p* < 0.005) (Figure 1A,D). When differentiating pleural effusions into early and late pleural effusions, we found that both early (*p* = 0.01) and late (*p* < 0.05) pleural effusions were associated with decreased lung allograft survival (Figure 1B,D). Importantly, associations between pleural effusion thoracocentesis and survival were assessed using time‐varying Cox regression analysis with the “status post thoracocentesis” as a time‐varying risk factor. Here, late pleural effusion thoracocentesis was associated with a higher hazard ratio (HR) for mortality (HR 4.00) compared to early pleural effusion thoracocentesis (HR 1.52) (Figure 1D). Exclusion of patients with (late) pleural effusion, thoracocentesis, and comorbid malignant disease (*n* = 5) did not affect this result. Patients who had early pleural effusion thoracocentesis were more likely to also have late pleural effusion thoracocentesis after LTX (Figure 1E, *p* < 0.001).

### 3.4 Pleural Effusions Requiring Thoracocentesis in LTX Recipients Are Associated With BLAD, but Not With CLAD

**FIGURE 2:**
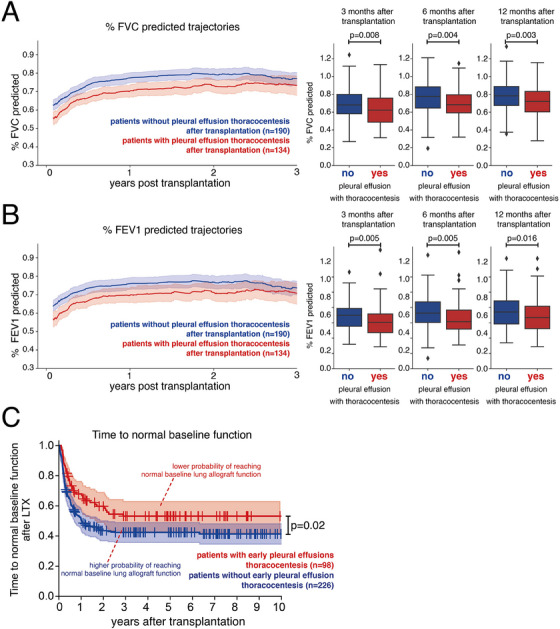
Pleural effusions requiring thoracocentesis are associated with BLAD, but not with CLAD. (A) The graph on the left shows the development of the average FVC% predicted with 95% confidence bands in LTX recipients with (red) and without (blue) pleural effusion thoracocentesis. The box plots on the right illustrate average FVC% predicted at 3, 6, and 12 months after transplantation in patients with (red) and without (blue) pleural effusion thoracocentesis. (B) The graph on the left shows the development of the average FEV1% predicted with 95% confidence bands in LTX recipients with (red) and without (blue) pleural effusion thoracocentesis. The Boxplots on the right illustrate average FEV1% predicted at 3, 6, and 12 months after transplantation in patients with (red) and without (blue) pleural effusion thoracocentesis (C) The Kaplan Meier Plot illustrates the time to normal baseline lung function in patients with (red) and without (blue) pleural effusion thoracocentesis after LTX. (A, B) Mann‐Whitney‐*U*‐Test and (C) Log‐Rank test.

Patients who required pleural effusion thoracocentesis showed significantly impaired lung function development after transplantation as seen for both FVC% (Figure 2A) and FEV1% (Figure 2B) at various timepoints after LTX, despite the successful treatment of the pleural effusions. LTX recipients who required pleural effusion thoracocentesis were more often affected by BLAD compared to patients who did not require pleural effusion thoracocentesis (55% vs. 45%, *p* = 0.04) (Figure S2). When differentiating between early and late pleural effusions after LTX, only early pleural effusions were significantly associated with BLAD (*p* = 0.05) (Figure S2A). A time‐to‐event analysis confirmed that patients with early pleural effusion thoracocentesis had a significantly lower probability of reaching normal baseline lung function (*p* = 0.02) (Figure 2C). Importantly, total pleural effusions as well as early and late pleural effusions were not associated with a diagnosis of CLAD (Figure S2B).

### 3.5 LTX Recipients With Obstructive Underlying Disease Are at Higher Risk of Early Pleural Effusions

Analyzing incidence of pleural effusions after LTX, we found a significantly lower freedom of thoracocentesis in LTX recipients with underlying obstructive disease (*p* = 0.02). This finding was mainly driven by an effect of early effusion thoracocentesis (*p* = 0.04), whereas there was no significant difference between patients with underlying interstitial or obstructive disease in regard to late pleural effusion thoracocentesis (*p* = 0.08). Previous publications [5, 12, 13] emphasized the role of the last actual measured recipient TLC before transplant (aTLC_recipient_) when performing donor‐recipient organ size mismatch analyses (Figure S3A, B). Patients transplanted for obstructive pulmonary diseases usually have a higher aTLC than pTLC due to lung hyperinflation, while patients with restrictive pulmonary diseases have a lower aTLC than pTLC due to fibrotic processes which both affect thorax size and rigidity and thus impact the selection of the donor lung (Figure S3C,D). We defined relative donor undersizing by pTLC_donor_/aTLC_recipient_ <0.8 and relative donor oversizing by pTLC_donor_/aTLC_recipient_ >1.2. Although donor‐recipient organ size mismatch is rare and small when comparing pTLC_donor_ and pTLC_recipient_ (classical donor recipient mismatch), *relative* donor undersizing is frequent in COPD (53%) while the same is true for *relative* donor organ oversizing in ILD (73%) (Figure S3C,D).

**FIGURE 3:**
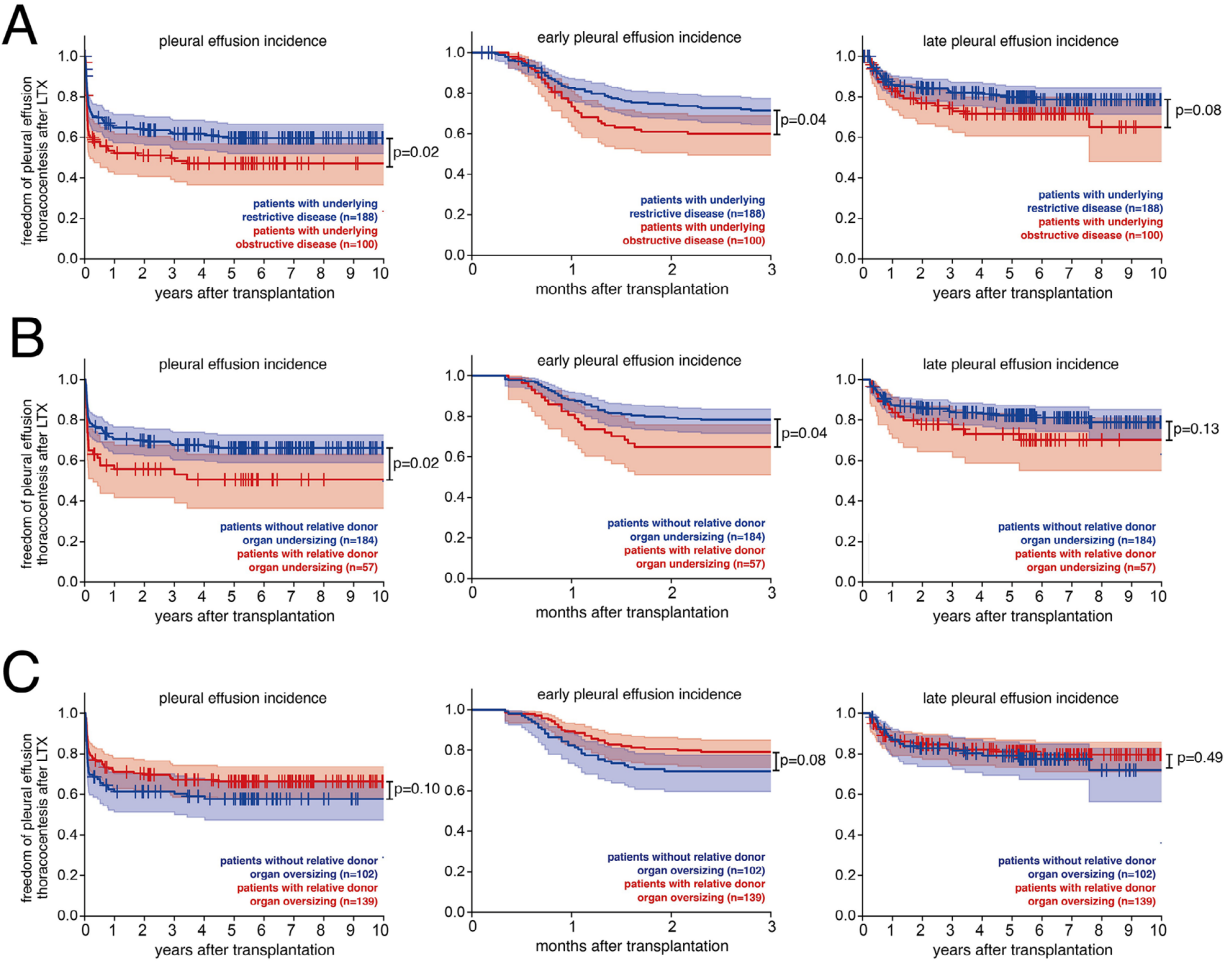
Patients with restrictive underlying disease and relative donor organ oversizing are at lower risk for early pleural effusions requiring thoracocentesis. (A) Kaplan Meier Plots depicting freedom from thoracocentesis over time for patients with underlying restrictive disease (blue) versus underlying obstructive disease (red) for all pleural effusions (left), for early pleural effusions (middle), for late pleural effusions (right). (B) Kaplan Meier Plots depicting freedom from thoracocentesis over time for patients without relative donor organ undersizing (blue) versus relative donor organ undersizing (red) for all pleural effusions (left), for early pleural effusions (middle), for late pleural effusions (right). (C) Kaplan Meier Plots depicting freedom from thoracocentesis over time for patients without relative donor organ oversizing (blue) versus relative donor organ oversizing (red) for all pleural effusions (left), for early pleural effusions (middle), for late pleural effusions (right). (A, B, C) Log‐Rank test.

As relative donor organ over‐ and undersizing were rare in patients with vascular, suppurative or other transplant indications, we analyzed the effects of the risk of relative donor size mismatching on pleural effusions for patients with underlying obstructive and restrictive disease only: Here, we found that relative donor undersizing substantially decreased the freedom of pleural effusion thoracocentesis in the total cohort (*p* = 0.02) as well as in the subgroup of early pleural effusions thoracocentesis (*p* = 0.04). Donor organ undersizing did not have a significant effect on late pleural effusion thoracocentesis (*p* = 0.13) (Figure 3B). Conversely, a similar trend was seen when analyzing donor organ oversizing: LTX recipients with relative donor organ oversizing had a longer freedom of thoracocentesis of pleural effusions (*p* = 0.10) (Figure 3C). This finding was seen for early effusions (*p* = 0.08), while there was no effect on late pleural effusions (*p* = 0.49) (Figure 3C).

### 3.6 LTX Recipients Undergoing Clamshell Thoracotomy Are at Higher Risk of Early Pleural Effusions

**FIGURE 4:**
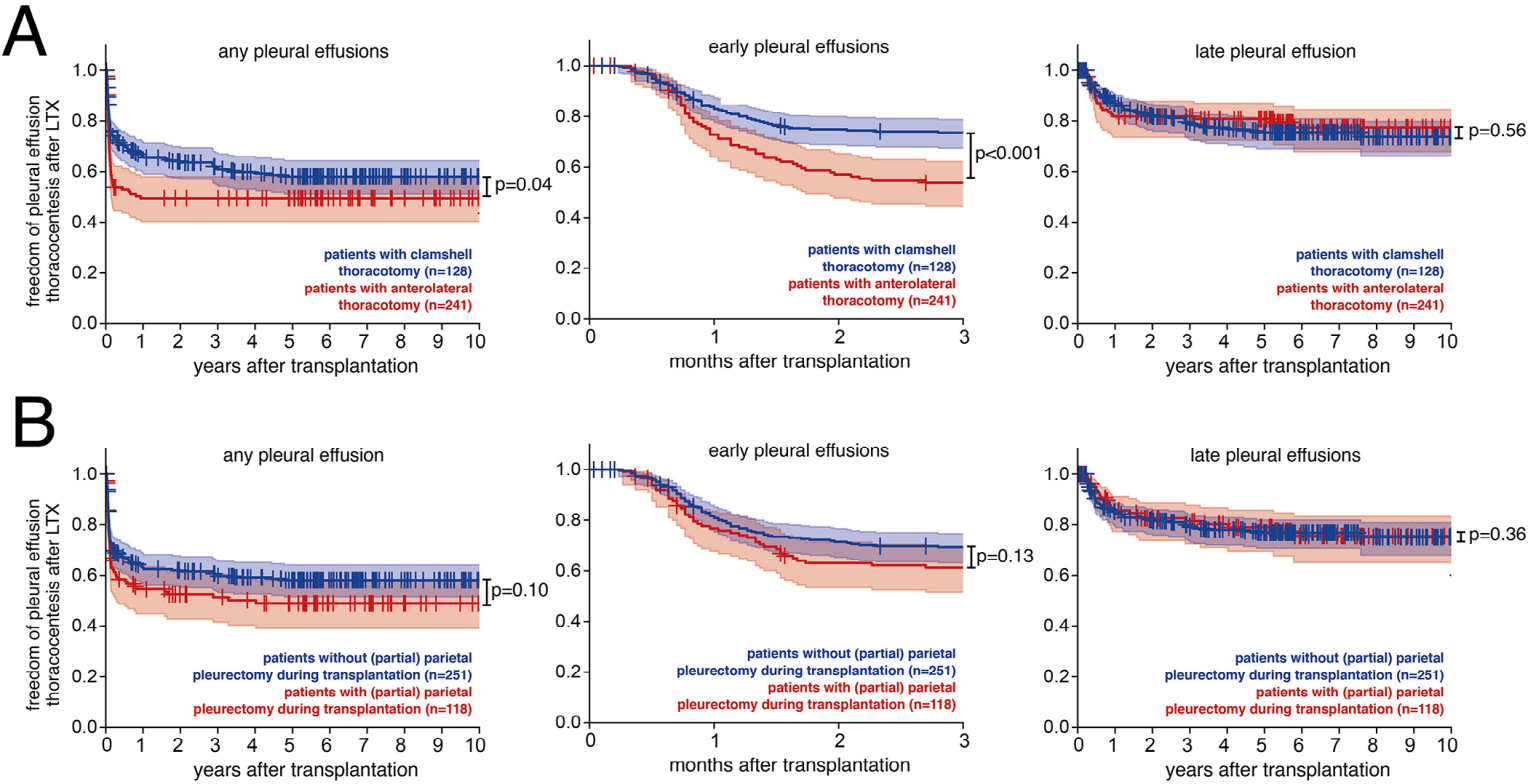
Patients undergoing clamshell surgery are at higher risk for early pleural effusions requiring thoracocentesis. (A) Kaplan Meier Plots depicting freedom from thoracocentesis over time for patients who had LTX surgery by clamshell thoracotomy (blue) versus anterior thoracotomy (red) for all pleural effusions (left), for early pleural effusions (middle) and for late pleural effusions (right). (B) Kaplan Meier Plots depicting freedom from thoracocentesis over time for patients who had LTX surgery involving a (partial) parietal pleurectomy (blue) versus without parietal pleurectomy (red) for all pleural effusions (left), for early pleural effusions (middle) and for late pleural effusions (right). (A, B) Log‐Rank test.

As early postoperative pleural effusions might be related to perioperative injury we analyzed if different surgical access routes impacted pleural effusion thoracocentesis. We found that patients who underwent clamshell thoracotomy had reduced freedom of pleural effusion thoracocentesis compared to patients with anterior thoracotomy (*p* = 0.04), which was only evident for early (*p* > 0.001) but not late pleural effusions (*p* = 0.56) (Figure 4A). Interestingly, injury to the parietal pleura in patients with compared to without pleurectomy did not affect freedom of either total, early, or late pleural effusions after LTX (Figure 4B).

## 4 Discussion

The pathophysiological origin of pleural effusions in LTX recipients, their risk factors and their subsequent impact on allograft function remains poorly understood. Here, we performed a retrospective, single‐center analysis focusing on pleural effusions and their associations with lung function trajectories, allograft dysfunction and allograft survival. We discovered a significant association of early pleural effusions with BLAD and further showed that pleural effusions were also associated with relative donor organ undersizing and COPD as underlying disease. Further, our study highlights the detrimental impact of early and late pleural effusions on long‐term survival of LTX recipients.

Recent analysis demonstrated that the majority of pleural effusions, both early and late after LTX, remain of unclear origin and that pleural effusions are associated with increased mortality [1, 2]. The best cut‐off point for time after LTX to differentiate between early and late pleural effusions is not clear, and different publications have used different thresholds [1, 2, 14]. Although both early and late pleural effusions are predominantly exudates, there appears to be a clear difference in regard to cellular composition: while early pleural effusions are neutrophil‐predominant exudates, late pleural effusions display a high proportion of lymphocytes [3, 14, 15]. In our analysis, several transplant‐related factors influenced freedom from early but not late pleural effusion thoracocentesis: donor lung undersizing, lower BMI, an underlying disease of COPD, and clamshell thoracotomy. The association between clamshell incision, which is usually performed for patients requiring ECMO, and early pleural effusion thoracocentesis may also reflect a subgroup of patients with greater disease severity. Altogether, this suggests that early pleural effusions may be more strongly correlated with transplant‐related factors and transplant‐related injury, whereas risk factors for late pleural effusions remain unknown. The only risk factor we could identify that was associated with late pleural effusion was a previous early pleural effusion.

Importantly, increased mortality was not associated with CLAD in our study, similar to other studies [1]. For early effusions, it seems plausible that the association with mortality might be mediated by BLAD. BLAD is a novel allograft dysfunction phenotype describing a failure to reach normal baseline lung function after LTX [4, 5]. Several centers have shown that BLAD affects more than one‐third of LTX recipients and conveys a strong mortality risk. Although a minority of patients reach normal baseline lung function with the first pulmonary function tests, the majority of patients start with subnormal lung function immediately after transplantation and improve over time [16]. Among others, BLAD has been associated with donor‐recipient size mismatch and pleural thickening [4, 5, 6, 17, 18]. Here, we were able to show that early pleural effusion thoracocentesis is a previously unrecognized and highly prevalent risk factor for BLAD. Of note, a consensus definition for BLAD has not been established so far. Recently, we have proposed a BLAD definition for SLTX recipients [4, 5]. In this manuscript, we performed the first analysis using a combination of the established DLTX and our proposed SLTX cut‐off in one analysis. As definitions may change in the future, this analysis thus has to be regarded with caution.

Patients with underlying obstructive lung disease were at greater risk of developing early pleural effusions; thus, part of the risk of developing an early pleural effusion might be mediated by relative donor undersizing. Convincingly, underlying restrictive lung disease and donor organ oversizing were associated with a lower risk of developing pleural effusions. Relative donor organ undersizing being a risk factor for early pleural effusions, but not late pleural effusions, suggests a distinct pathophysiological process. When analyzing the effect of pleural effusion thoracocentesis on BLAD in separate subgroups, similar effects were seen both for DLTX and SLTX cohorts (data not shown), strengthening both the proposed cut‐offs as well as the effects of pleural effusion thoracocentesis as a risk factor for BLAD.

However, the reason why late pleural effusions are strongly associated with mortality remains unknown, and according to our data, this does not seem to be an effect mediated by BLAD or malignancy. In other studies, the etiology of late pleural effusions remained unclear in the majority of cases [1]. It is possible that the predominance of lymphocytes in these effusions indicates an active (inflammatory) process at the interface of parietal (recipient) pleura and visceral (donor) pleura. Importantly, in our cohort, there was a high incidence of pleural effusion thoracocentesis, with around 50% of patients ever receiving thoracocentesis, which underlines the general importance and frequency of this problem. In the publication by Joean et al. and Tang et al. incidence of pleural effusions after LTX was lower, with around 10% and 26%, respectively, which might be attributed to different thoracocentesis strategies/thresholds of the treating transplant physicians [1, 2].

In this retrospective setting, we chose to analyze only pleural effusions that required thoracocentesis, as this provided a clear and timely well‐defined endpoint. Thus, we avoided challenges with rather vague endpoints like occurrence or resolution of pleural effusion, which might differ between imaging modalities and might introduce further bias due to a variability in frequency and indication for thoracic imaging among patients. It is conceivable that underlying abnormalities in the pleura or pleural mechanics that lead to pleural effusion formation remain and limit normal development, especially in the early time after LTX, where lung function is still increasing. Consistent with this idea is the finding of a study that showed pleural thickening upon CT scans in a majority of LTX recipients 12 months after LTX [18].

We believe this study is of importance as it confirms previous findings of early and late pleural effusions obtained by other groups, emphasizes the strong association of pleural effusions with mortality and describes for the first time a strong association of early pleural effusions with BLAD. Although this study included patients transplanted between 2010 and 2018 to ensure proper follow‐up time in all patients, recent alterations in post LTX care might potentially alter the significance of pleural effusions in recent LTX recipients. Another limitation of this manuscript is the retrospective and single‐center design. As such, indication for pleural effusion drainage and the subsequent diagnostic pleural effusion workup were at the discretion of the treating physician. In this regard, data on serum and pleural chemistry as well as pleural cytology were inhomogeneous and could not be thoroughly analyzed.

We believe that further research into pathophysiology and the deleterious outcomes of LTX recipients with pleural effusions is urgently needed. As such, we have initiated a prospective pleural effusion biobanking of LTX recipients at our center that will provide a more comprehensive insight into specific pathophysiologic drivers of pleural abnormalities and pleural effusion development and their contribution to lung function impairment in LTX recipients in the near future.

## Author Contributions

Conception of the study: Michael Gerckens, Nikolaus Kneidinger, and Carlo Mümmler Data acquisition and patient treatment: Michael Gerckens, Nicole Weiss, Daria Khmelovska, Alexander Richard, Mathias Klemm, Philipp Plohmann, Paola Arnold, Tobias Veit, Jürgen Barton, Teresa Kauke, Christian Schneider, Sebastian Michel, Michael Irlbeck, Ali Önder Yildirim, Jürgen Behr, Nikolaus Kneidinger, and Carlo Mümmler Data analysis and interpretation: Michael Gerckens, Nicole Weiss, Daria Khmelovska, Alexander Richard, Nikolaus Kneidinger, and Carlo Mümmler Manuscript draft: Michael Gerckens, Nicole Weiss, Daria Khmelovska, Alexander Richard, Mathias Klemm, Philipp Plohmann, Paola Arnold, Tobias Veit, Jürgen Barton, Teresa Kauke, Christian Schneider, Sebastian Michel, Michael Irlbeck, Ali Önder Yildirim, Jürgen Behr, Nikolaus Kneidinger, and Carlo Mümmler

## Conflicts of Interest

The authors declare no conflicts of interest.

## Supporting information




**Figure S1**: Study flow chart
**Figure S2**: Pleural effusions are associated with BLAD, but not with CLAD.
**Figure S3**: Relative donor organ undersizing due to thorax expansion increases the risk of pleural effusions.

## Data Availability

The data analyzed during the current study are not publicly available but are available from the corresponding author on reasonable request.
