# Supplementary material for: Pleural Effusions Requiring Thoracocentesis Are Associated With Baseline Lung Allograft Dysfunction and Mortality in Lung Transplant Recipients
Source: Clin Transplant. 2025 Aug 22;39(8):e70234. doi: 10.1111/ctr.70234 (PMC12372864; doi:10.1111/ctr.70234)
Supplement: Supplementary file 1 — Figure S1: Study flow chart Figure S2: Pleural effusions are associated with BLAD, but not with CLAD. Figure S3: Relative donor organ undersizing due to thorax expansion increases the risk of pleural effusions. [file CTR-39-e70234-s001.docx]

**SUPPLEMENTARY FIGURES WITH FIGURE LEGENDS**

**Figure S1: Study flow chart**

**
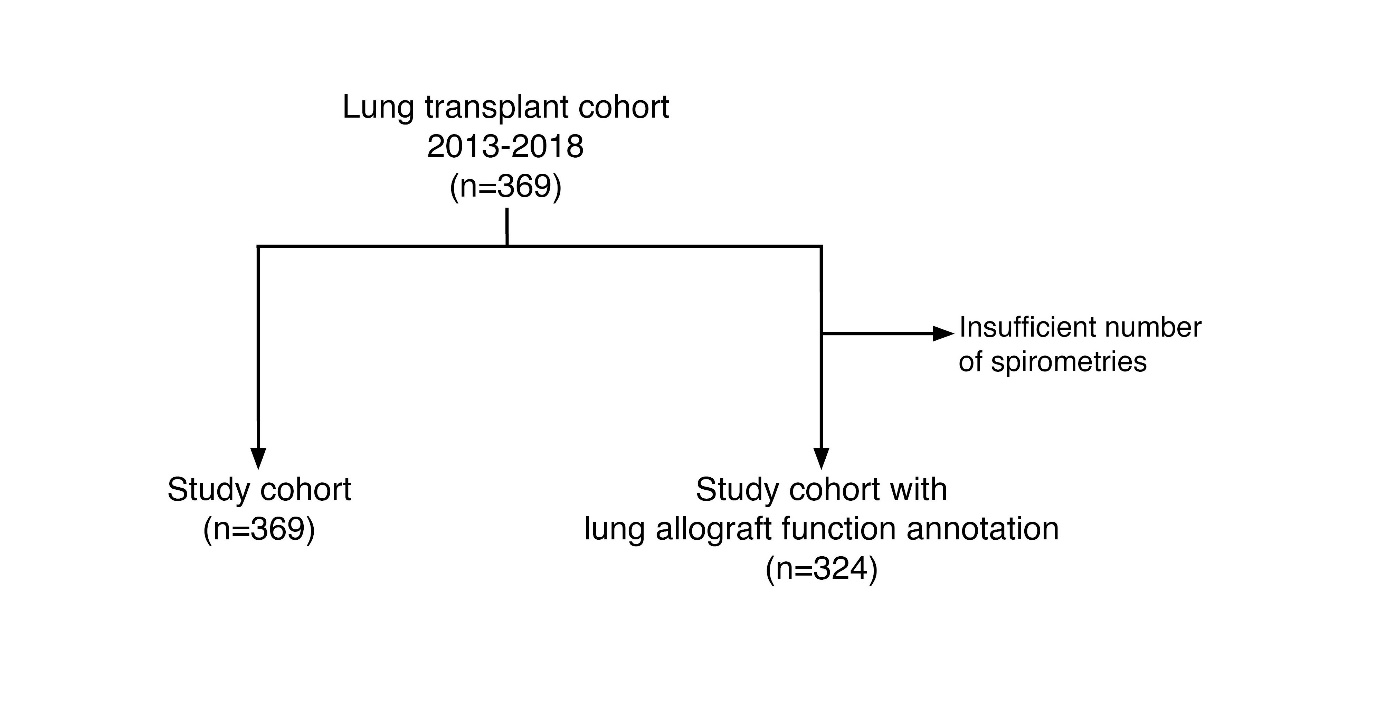
**

**Figure S1:** Study flowchart illustrating patient cohort selection.

**Figure S2: Pleural effusions are associated with BLAD, but not with CLAD.**


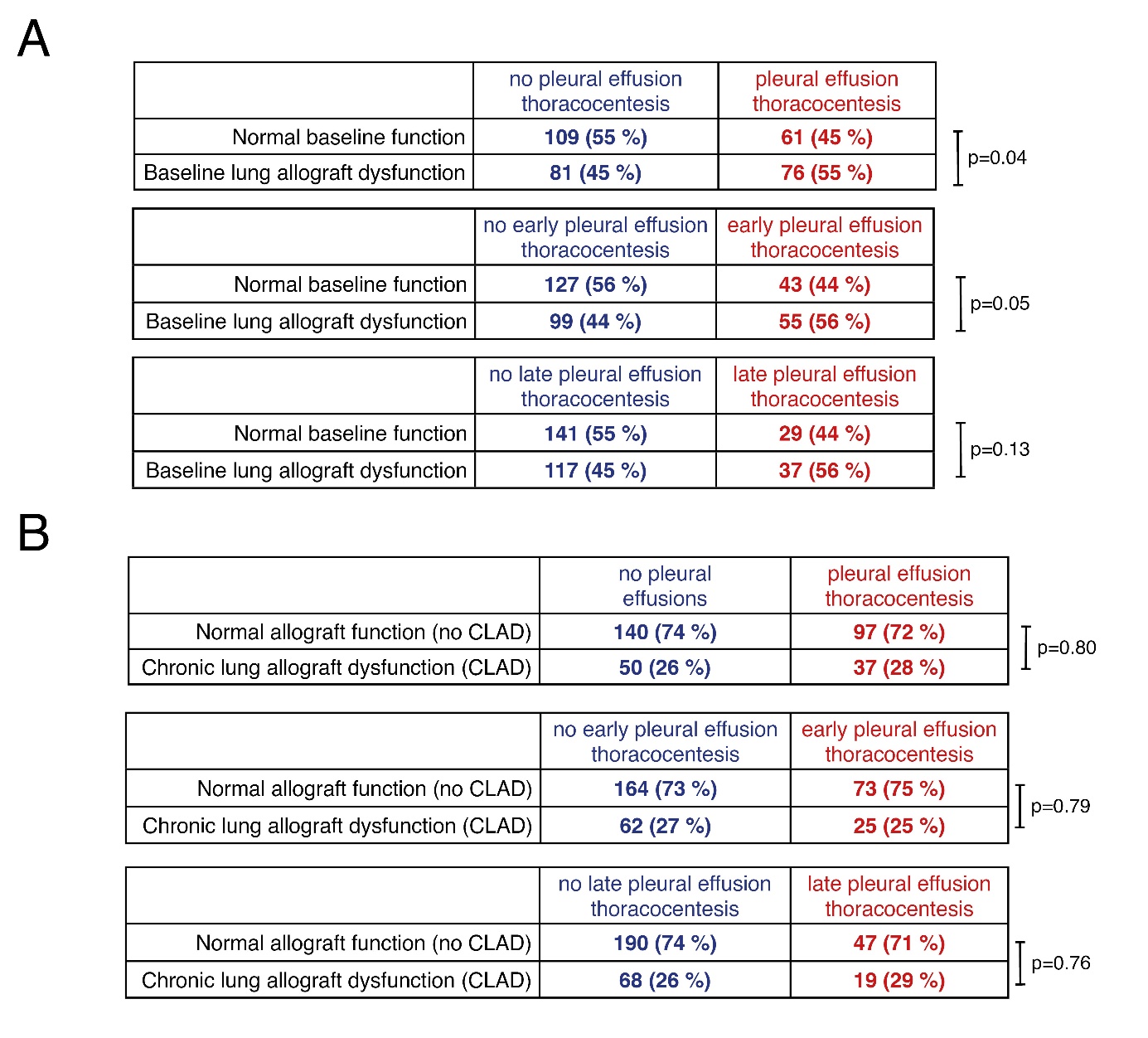


**Figure S2:** Contingency tables, displayed as cross tables, illustrating associations of early and late pleural effusions with **(A)** baseline lung allograft dysfunction and **(B)** chronic lung allograft dysfunction. Statistics: (A, B) Fisher exact test.

**Figure S3: Relative donor organ undersizing due to thorax expansion increases the risk of pleural effusions.**


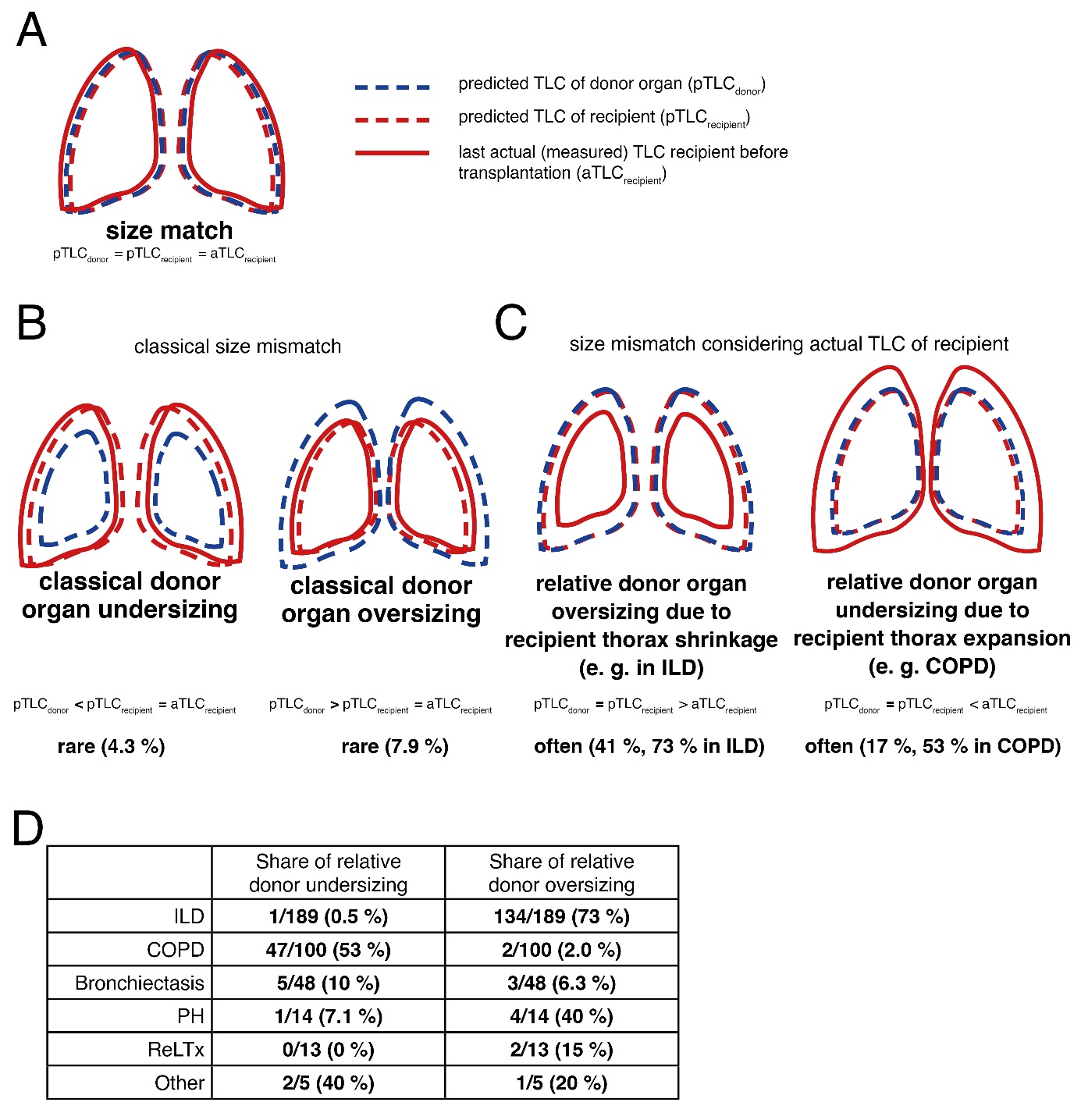


**Figure S3:** Size mismatching in lung transplant recipients. Illustration explaining (A) size match in contrast to (B) classical and (C) relative size mismatching. In (D) the proportion of relative donor size mismatching according to different underlying diseases in our cohort is illustrated**.** Abbreviations: aTLC: actual total lung capacity; pTLC: predicted total lung capacity.
